# Structural modeling of tissue-specific mitochondrial alanyl-tRNA synthetase (*AARS2*) defects predicts differential effects on aminoacylation

**DOI:** 10.3389/fgene.2015.00021

**Published:** 2015-02-06

**Authors:** Liliya Euro, Svetlana Konovalova, Jorge Asin-Cayuela, Már Tulinius, Helen Griffin, Rita Horvath, Robert W. Taylor, Patrick F. Chinnery, Ulrike Schara, David R. Thorburn, Anu Suomalainen, Joseph Chihade, Henna Tyynismaa

**Affiliations:** ^1^Research Programs Unit, Molecular Neurology, Biomedicum Helsinki, University of HelsinkiHelsinki, Finland; ^2^Department of Clinical Chemistry, University of Gothenburg, Sahlgrenska University HospitalGothenburg, Sweden; ^3^Department of Pediatrics, Queen Silvia Children’s Hospital, University of GothenburgGothenburg, Sweden; ^4^Institute of Genetic Medicine, Wellcome Trust Centre for Mitochondrial Research, Newcastle UniversityNewcastle upon Tyne, UK; ^5^Institute of Neuroscience, Wellcome Trust Centre for Mitochondrial Research, Newcastle UniversityNewcastle upon Tyne, UK; ^6^Department of Neuropediatrics, Developmental Neurology and Social Pediatrics, University of EssenEssen, Germany; ^7^Murdoch Childrens Research Institute, Royal Childrens Hospital and Department of Paediatrics, University of MelbourneMelbourne, VIC, Australia; ^8^Department of Neurology, Helsinki University Central HospitalHelsinki, Finland; ^9^Department of Chemistry, Carleton CollegeNorthfield, MN, USA; ^10^Department of Medical Genetics, Haartman Institute, University of HelsinkiHelsinki, Finland

**Keywords:** mitochondrial disease, aminoacyl-tRNA synthetases, alanyl-tRNA synthetase, tissue-specificity, structural modeling

## Abstract

The accuracy of mitochondrial protein synthesis is dependent on the coordinated action of nuclear-encoded mitochondrial aminoacyl-tRNA synthetases (mtARSs) and the mitochondrial DNA-encoded tRNAs. The recent advances in whole-exome sequencing have revealed the importance of the mtARS proteins for mitochondrial pathophysiology since nearly every nuclear gene for mtARS (out of 19) is now recognized as a disease gene for mitochondrial disease. Typically, defects in each mtARS have been identified in one tissue-specific disease, most commonly affecting the brain, or in one syndrome. However, mutations in the *AARS2* gene for mitochondrial alanyl-tRNA synthetase (mtAlaRS) have been reported both in patients with infantile-onset cardiomyopathy and in patients with childhood to adulthood-onset leukoencephalopathy. We present here an investigation of the effects of the described mutations on the structure of the synthetase, in an effort to understand the tissue-specific outcomes of the different mutations. The mtAlaRS differs from the other mtARSs because in addition to the aminoacylation domain, it has a conserved editing domain for deacylating tRNAs that have been mischarged with incorrect amino acids. We show that the cardiomyopathy phenotype results from a single allele, causing an amino acid change R592W in the editing domain of *AARS2*, whereas the leukodystrophy mutations are located in other domains of the synthetase. Nevertheless, our structural analysis predicts that all mutations reduce the aminoacylation activity of the synthetase, because all mtAlaRS domains contribute to tRNA binding for aminoacylation. According to our model, the cardiomyopathy mutations severely compromise aminoacylation whereas partial activity is retained by the mutation combinations found in the leukodystrophy patients. These predictions provide a hypothesis for the molecular basis of the distinct tissue-specific phenotypic outcomes.

## INTRODUCTION

Mitochondrial protein synthesis produces key subunits of the oxidative phosphorylation (OXPHOS) complexes that generate the majority of the ATP for our cells. The protein synthesis in mitochondria requires careful synchrony between two genomes and cellular compartments, because the protein components of its machinery are encoded by the nuclear genome and imported into mitochondria, whereas the RNA components (2 rRNAs and 22 tRNAs) are encoded by mitochondrial DNA (Hallberg and Larsson, 2014). Among the necessary proteins are the aminoacyl-tRNA synthetases (ARSs) that recognize specific tRNAs and charge them with cognate amino acids, thus contributing to the initiation and accuracy of the protein synthesis. Nineteen ARSs function in mammalian mitochondria (mtARSs), one for each amino acid, except glutamine, which is charged by an alternative pathway (Nagao et al., 2009).

Defects of mitochondrial translation are a major cause of diseases that lead to mitochondrial OXPHOS dysfunction (Rotig, 2011). Since 2007, the mtARS genes have been recognized as a new group of disease genes affecting mitochondrial protein synthesis (Konovalova and Tyynismaa, 2013; Diodato et al., 2014a; Schwenzer et al., 2014). Proteins need to be synthesized in mitochondria in all cell types, with the exception of red blood cells, and thus the tissue-specificity of the phenotypes caused by mutations in the different mtARS genes has been unexpected. Now that pathogenic mutations have been reported in 15 genes encoding mtARSs (and in the two genes *GARS* and *KARS* encoding dual localized synthetases for cytoplasmic and mitochondrial translation), we can, however, conclude that the majority of the mtARS defects affect the central nervous system. Nevertheless, the range of CNS phenotypes is also remarkably wide, affecting specific neurons in many cases, and including patients with infantile-, early- and late-onset of disease.

Central nervous system involvement as the main or sole clinical presentation is found in defects of the mtARSs for aspartate (*DARS2*; Scheper et al., 2007), glutamate (*EARS2*; Steenweg et al., 2012), methionine (*MARS2*; Bayat et al., 2012), phenylalanine (*FARS2*; Elo et al., 2012), arginine (*RARS2*; Edvardson et al., 2007), valine (*VARS2*; Diodato et al., 2014b), threonine (*TARS2*; Diodato et al., 2014b), proline (*PARS2*; Sofou et al., 2015), asparagine (*NARS2*; Sofou et al., 2015), and cysteine (*CARS2*; Hallmann et al., 2014). Exceptions are the mutations in the genes for histidyl-tRNA (*HARS2*) and leucyl-tRNA (*LARS2*) synthetases that cause sensorineural hearing loss and ovarian dysgenesis (Pierce et al., 2011, 2013), for tyrosyl-tRNA synthetase (*YARS2*) in a combination of myopathy and anemia (Riley et al., 2010), and for seryl-tRNA synthetase (*SARS2*; Belostotsky et al., 2011) in HUPRA, for hyperuricemia, pulmonary hypertension, renal failure in infancy, and alkalosis, syndrome. A common feature for all the defects mentioned above is that so far each of them has been described in one tissue-specific phenotype or in one syndrome.

We identified mutations in the gene for mitochondrial alanyl-tRNA synthetase (*AARS2*) in a phenotype that differs from the other reported mtARS defects, being clinically a fatal early onset cardiomyopathy (Gotz et al., 2011). Our patients died within the first 10 months of life with severe cardiomyopathy, having a significant reduction in OXPHOS complexes in the heart and partially also in the skeletal muscle and brain. Two other studies have later reported patients with fatal infantile-onset *AARS2* mutations (Calvo et al., 2012; Taylor et al., 2014). Although cardiomyopathy is not a usual feature of the other mtARS defects, it is typical in early infancy in many other mitochondrial protein synthesis defects, such as in those caused by mutations in genes encoding for mitoribosome subunits *MRPL44* and *MRPL3* (Galmiche et al., 2011; Carroll et al., 2013), in tRNA-modifiers *MTO1* (Ghezzi et al., 2012) and *GTPBP3* (Kopajtich et al., 2014), and in RNA processing enzyme *ELAC2* (Haack et al., 2013). Remarkably, a recent study reported *AARS2* mutations in patients who developed leukoencephalopathy and ovarian failure, with onset from childhood to adulthood, and no signs of a cardiomyopathy (Dallabona et al., 2014). *AARS2* has thus been an example of mutations in the same mtARS gene leading to two very different diseases with dissimilar tissue involvement.

The mitochondrial alanyl-tRNA (mtAlaRS) synthetase encoded by *AARS2* differs itself from the other mtARSs, because it has in addition to the aminoacylation domain a conserved editing domain for deacylating mischarged tRNAs (Beebe et al., 2003; Guo et al., 2009b; Gotz et al., 2011). The mischarging results from the inability of the enzyme’s aminoacylation domain to discriminate alanine from two other small amino acids, serine and glycine. In addition, the synthetase has a C-terminal domain, which is required for cooperative binding of both the aminoacylation and editing domains to the tRNA (Guo et al., 2009a). We have previously hypothesized that in our cardiomyopathy patients, the *AARS2* mutation resulting in R592W change, which is located in the editing domain of the synthetase, might interfere with tRNA binding, thus preventing editing, and resulting in mistranslation (Gotz et al., 2011). Based on homology modeling, we now present structural predictions of all *AARS2* missense mutations in the two different phenotypes, and show that the tissue-specific defects affect distinct domains of the synthetase but compromise the aminoacylation with different outcomes. Furthermore, we show that the *AARS2* cardiomyopathy mutation (R592W) is a common founder mutation and carried by all the identified patients with the severe infantile-onset phenotype.

## MATERIALS AND METHODS

### HAPLOTYPE ANALYSIS

Nine single-nucleotide polymorphisms (SNPs) flanking *AARS2* gene were selected for haplotype analysis, based on the haplotype blocks presented previously (Taylor et al., 2014). Genomic DNA was amplified by PCR using flanking oligonucleotides (sequences available on request) and Sanger sequenced.

### STRUCTURE PREDICTION

For human mtAlaRS homology modeling we used the recently solved structure of full-length alanyl-tRNA synthetase (AlaRS) from *Archaeoglobus fulgidus* as a template. This bacterial AlaRS is a homodimer with one subunit crystallized with bound tRNA substrate and an alanyl-adenylate analog in so-called “closed” conformation, while the second subunit is in “open,” substrate free conformation (Naganuma et al., 2014; PDB id 3WQY). “Closed” conformation of human mtAlaRS was modeled using A chain in solved *A. fulgidus* AlaRS structure (3WQY_A), and the “open” substrate free form was modeled using B chain of the same structure (3WQY_B). Sequence alignment of human mtAlaRS (985 aa) and template *A. fulgidus* AlaRS (906 aa) was done using similarity matrix BLOSUM62. Multiple sequence alignments of human mtAlaRS and template sequence with a number of prokaryote and eukaryote homologs were done using PROMALS3D server. Obtained alignment was then submitted for homology modeling to the SWISS-MODEL server. Modeling was performed using alignment mode using either 3WQY_A or 3WQY_B structure as template. Resulting “open” and “closed” structures were analyzed using Discovery Studio v4.1 (Accelrys) software. Overall architecture and arrangement of secondary structure elements in the obtained models was similar to those in the template structures except for two loops, encompassing residues 211–231 and 540–561, which were omitted in the analysis. Docking of the bacterial tRNA^Ala^ and alanyl-adenylate into the model was done after superimposition of template chain A with the modeled human mtAlaRS using Discovery Studio v4.1 (Accelrys) software.

## RESULTS

### THE *AARS2* c.1774C > T (R592W) IS A FOUNDER MUTATION AND CARRIED BY EVERY PATIENT WITH THE CARDIOMYOPATHY PHENOTYPE

Seven patients with infantile-onset cardiomyopathy and *AARS2* mutations have been reported (Gotz et al., 2011; Taylor et al., 2014) and one who died in utero was described to have myopathy, hypotonia and multiple fractures (Calvo et al., 2012). In addition, we report here three additional patients of Swedish or German origin.

The Swedish patient was a boy born as the second child of healthy, non-consanguineous parents. An older sibling was healthy. The pregnancy and delivery were normal. Birth weight was 3990 g. The Apgar Scores were 2, 5, and 7 at 1, 5, and 10 min. At birth the boy was floppy, had decreased movements and presented breathing difficulties needing ventilatory treatment from the age of 1 h. He was diagnosed with a hypertrophic cardiomyopathy involving both the left and right ventricles, and with lactic acidosis. He was referred for further investigation at the age of 13 days. A skeletal muscle biopsy performed at the age of 14 days showed a mitochondrial myopathy with ragged red fibers, which were cytochrome-c oxidase negative, and with increased lipid droplets. Investigation of isolated skeletal muscle mitochondria showed decreased activities of complexes I-IV. The cardiomyopathy was progressive and the boy died at the age of 7 weeks of cardio-respiratory failure. An autopsy was performed and showed a severely hypertrophic heart that weighed 60 g.

The German patients were twin girls of healthy, non-consanguineous parents. The pregnancy and delivery were normal. Birth weights of the first and second twin were 1890 g and 2150 g, respectively. The Apgar Scores were 9, 10, 10, and 6, 8, 9, respectively. Onset of disease in the first twin was at birth with breathing difficulties needing ventilatory treatment from the age of 8 h. The second twin was stable and did not need ventilatory support. The first twin was diagnosed with a hypertrophic cardiomyopathy involving mostly the left ventricle and with lactic acidosis at the age of 8 weeks. A skeletal muscle biopsy performed at the age of 11 weeks showed a mitochondrial myopathy with cytochrome-c oxidase negative fibers and perimysial fibrosis as well as reduced activity of coenzyme Q. Investigation of respiratory chain complexes disclosed reduced activities for complexes I–IV. The cardiomyopathy was progressive and the girl died at the age of 16 weeks of cardio-respiratory failure. The symptoms in the second twin started a few weeks later and showed the same clinical course, she died at the age of 20 weeks.

Out of these eleven patients six were homozygous for the c.1774C > T (R592W) mutation and five had compound heterozygous *AARS2* mutations with each of them carrying the same c.1774C > T (R592W) mutation in one allele (**Table 1**).

**Table 1:** List of all patients reported with mutations in *AARS2* that presented with infantile-onset cardiomyopathy.

Mutation (s)	Country of origin	Age at death	Clinical presentation	Reference
**Homozygous**
R592W	Finnish	10 months	cardiomyopathy, muscle, CNS	Gotz et al. (2011)
R592W	German	4–5 months (twins)	cardiomyopathy, muscle	This report
R592W	Swedish	7 weeks	cardiomyopathy, muscle	This report
R592W	German	2 months	cardiomyopathy, muscle	Taylor et al. (2014)
R592W	British	11 months	cardiomyopathy	Taylor et al. (2014)
**Compound heterozygous**
R592W, L155R	Finnish	3 days (sibling died in utero)	Cardiomyopathy, muscle, CNS	Gotz et al. (2011)
R592W, R329H	Australian	in utero	Stillborn fetus, muscle	Calvo et al. (2012)
R592W, A961V	British	1.5 months	Cardiomyopathy, muscle, CNS	Taylor et al. (2014)
R592W, C218Lfs*6	British	3 months	Cardiomyopathy	Taylor et al. (2014)
R592W, Y539C	German	1 month	Cardiomyopathy, muscle, CNS	Taylor et al. (2014)

*All patients were either homozygous or compound heterozygous for c.1774C > T (R592W) mutation. Clinical description regarding muscle and central nervous system (CNS) involvement is included*.

**FIGURE 1:**
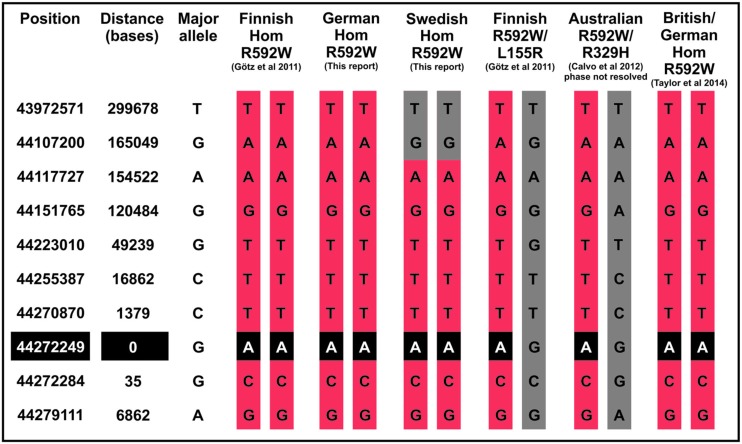
**Patient haplotypes around the *AARS2* mutation.** The haplotype of the Swedish patient shows recombination 165 kilobases upstream of the mutation site.

Patients with British or German origin were recently suggested to share a common disease haplotype for R592W (Taylor et al., 2014). We present here the haplotypes surrounding this mutation for patients from Finland, Sweden, and Australia in comparison to the British and German patients, which suggest that the shared mutation described in each *AARS2* cardiomyopathy patient originates from a common founder (**Figure 1**).

Carriers of the c.1774C > T (R592W) mutation can be found in exome sequencing databases of different populations (**Table 2**), with the highest minor allele frequency of 0.0006 in Finland, where the first *AARS2* cardiomyopathy patients were identified.

**Table 2:** Allele frequency of the mutation leading to amino acid change R592W in different exome/genome databases. SISU and 1000 Genomes contribute to the larger ExAC database.

**Database**	**Total alleles**	**Mutation alleles**	**Minor allele frequency**
1000 Genomes (1000 genomes.org)	5008	1	0.0002

SISU (sisu.fimm.fi)	6646	4	0.0006

EVS (evs.gs.washington.edu)	13006	3	0.0002

ExAC (exac.broadinstitute.org)	126272	35	0.0003

### THE DISTRIBUTION OF THE MUTATED AMINO ACIDS IN CARDIOMYOPATHY AND LEUKODYSTROPHY SUGGESTS DIFFERENTIAL MOLECULAR PATHOGENESIS

**FIGURE 2:**
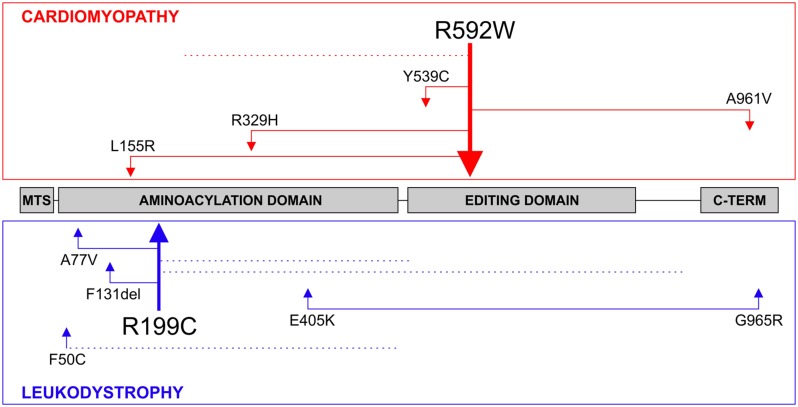
***AARS2* mutations.** The compound heterozygous mutations found in patients with cardiomyopathy or leukodystrophy are illustrated. Truncating mutations that are predicted to destabilize the entire synthetase are marked with dotted lines. The thicker arrows demonstrate recurring mutations. The *AARS2* mutation leading to the R592W amino acid change has been the only one identified in a homozygous state.

The R592W amino acid change locates to the editing domain of the mtAlaRS and we initially hypothesized that it causes mitochondrial mistranslation, which can be expected to harm OXPHOS assembly and function by amino acid misincorporation in the subunits that are synthesized by mitoribosomes (Gotz et al., 2011). The new *AARS2* mutations reported in patients with leukodystrophy seemed, at first glance, to be scattered along the entire *AARS2* gene, with some located in the aminoacylation domain and some in the editing domain (Dallabona et al., 2014). All of the leukodystrophy patients had compound heterozygous mutations. To clarify the range and localization of the mutations in both phenotypes, we analyzed them in the actual combinations in which they were found in the patients (**Figure 2**). The cardiomyopathy patients were either homozygous for the editing domain mutation R592W or had it in combination with a mutation located in the aminoacylation, editing or the C-terminal domain or with a truncating mutation. Mutations that cause premature termination or frameshift can be expected to be total loss-of-function alleles with no stable synthetase present. The mutation spectrum was very different for the leukodystrophy patients because these presented combinations of two missense mutations in the aminoacylation domain or an aminoacylation domain missense mutation with a truncating mutation, with one exception, which was a combination of missense mutations in the aminoacylation domain and the C-terminal domain. Thus none of the missense mutations in leukodystrophy patients were in the editing domain, which was in striking contrast to all of the cardiomyopathy patients (**Figure 2**).

### OVERVIEW OF THE STRUCTURAL MODEL OF HUMAN mtAlaRS

**FIGURE 3:**
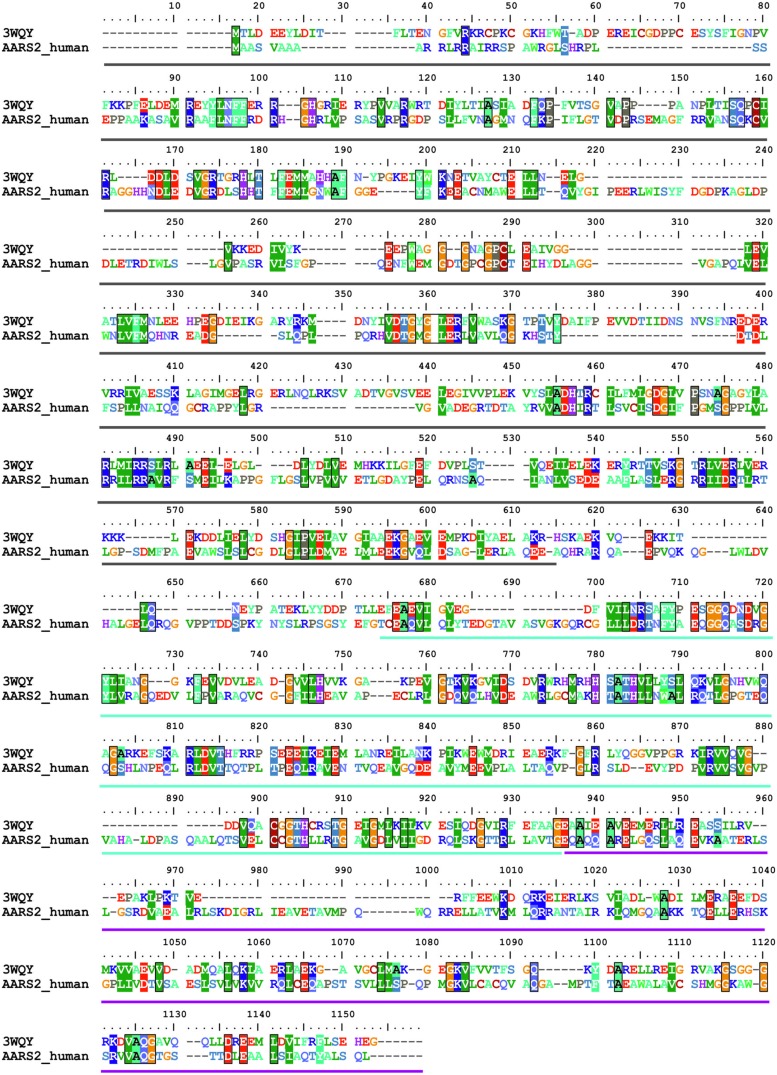
**Sequence alignment between human mtAlaRS and AlaRS from *A. fulgidus* (3WQY).** The alignment was extracted from multiple sequence alignment of AlaRS homologs from eukaryotes and prokaryotes using BioEdit software. The aminoacylation domain is marked with dark gray bar, the editing domain with cyan, and the C-terminal domain with magenta bars. Identical/similar amino acid residues in the same position are marked with colored boxes.

**FIGURE 4:**
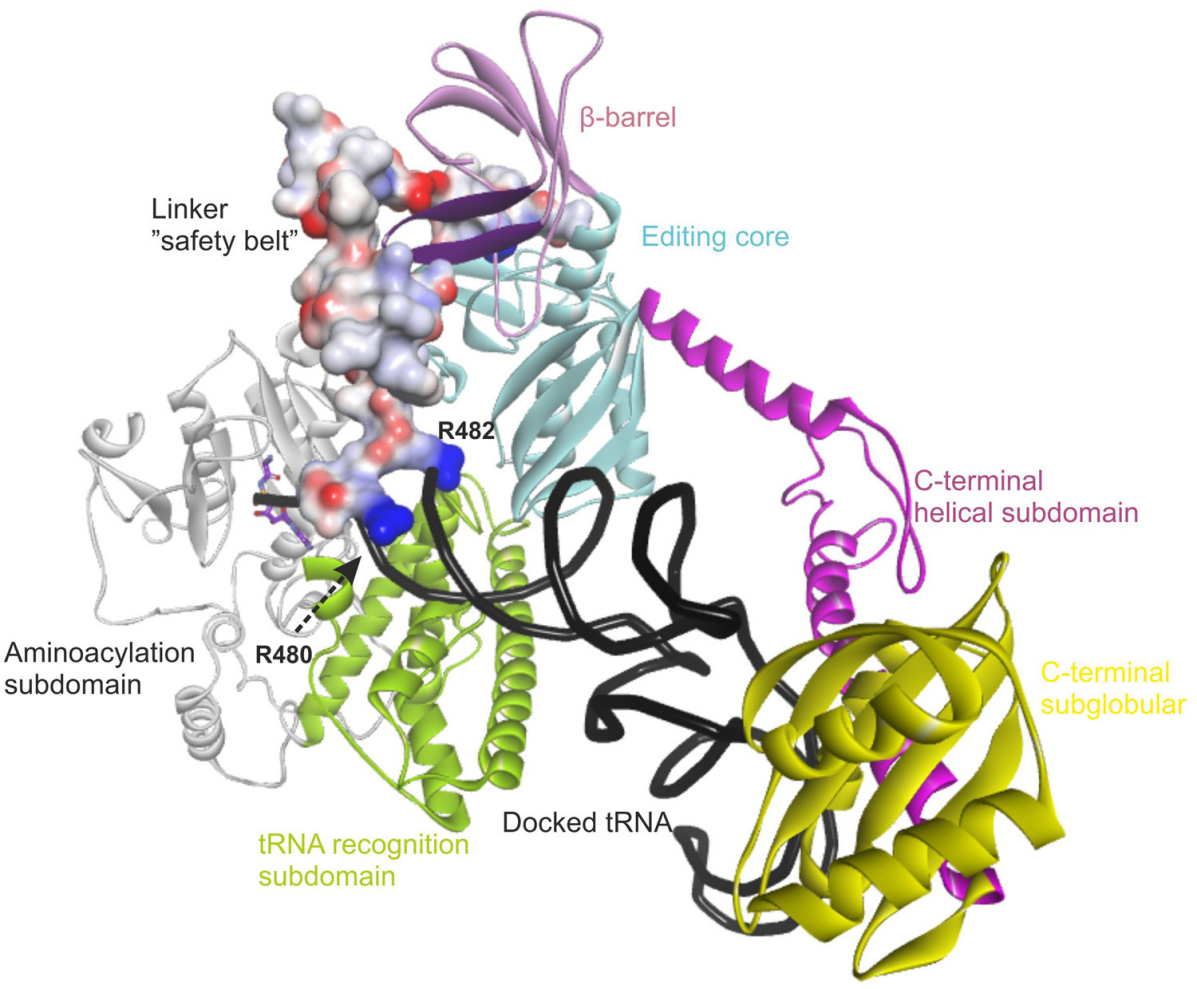
**Overview of the modeled structure of human AlaRS.** Aminoacylation subdomain (24–312 aa) is in silver, tRNA recognition subdomain (313–477 aa) of aminoacylation domain is in green, linker (“safety belt” – 478–529 aa) between tRNA recognition and editing domain is presented as solvent interpolated charge surface, β-barrel (530–621 aa) and editing core (622–783 aa) subdomains of editing domain are in pink and cyan, respectively. C-Ala domain or C-terminal domain is predicted to have helical (784–874 aa in magenta) and globular (875–985 aa in yellow) subdomains. R480 and R482 from the linker are predicted to stabilize bound tRNA within the aminoacylation domain. Backbone of docked tRNA is shown in dark gray. Docked alanyl-adenylate in the aminoacylation site is shown as stick model with carbon atoms labeled with magenta. Part of β-barrel subdomain (592–604 aa) of the editing domain proposed to interact with the linker is marked with purple (see text).

The recent crystal structure of a full-length length AlaRS (Naganuma et al., 2014) made possible our current complete structural model of the human mtAlaRS, which includes the arrangement of all three domains: aminoacylation, editing and C-terminal. The human mtAlaRS and template *A. fulgidus* AlaRS sequences shared overall 17.5% identity and 30.9% similarity (**Figure 3**). Domain-specific sequence identity and similarity was 17.5 and 31.2% for the aminoacylation domain, 25.0 and 39.8% for the editing domain and 14.7 and 26.5% for the C-terminal domain, respectively. Each domain can be further divided into two subdomains: the aminoacylation domain contains subdomains for aminoacylation and tRNA recognition, the editing domain consists of β-barrel and editing core, and the C-terminal or so-called C-Ala domain consists of helical and globular subdomains (**Figure 4**).

**FIGURE 5:**
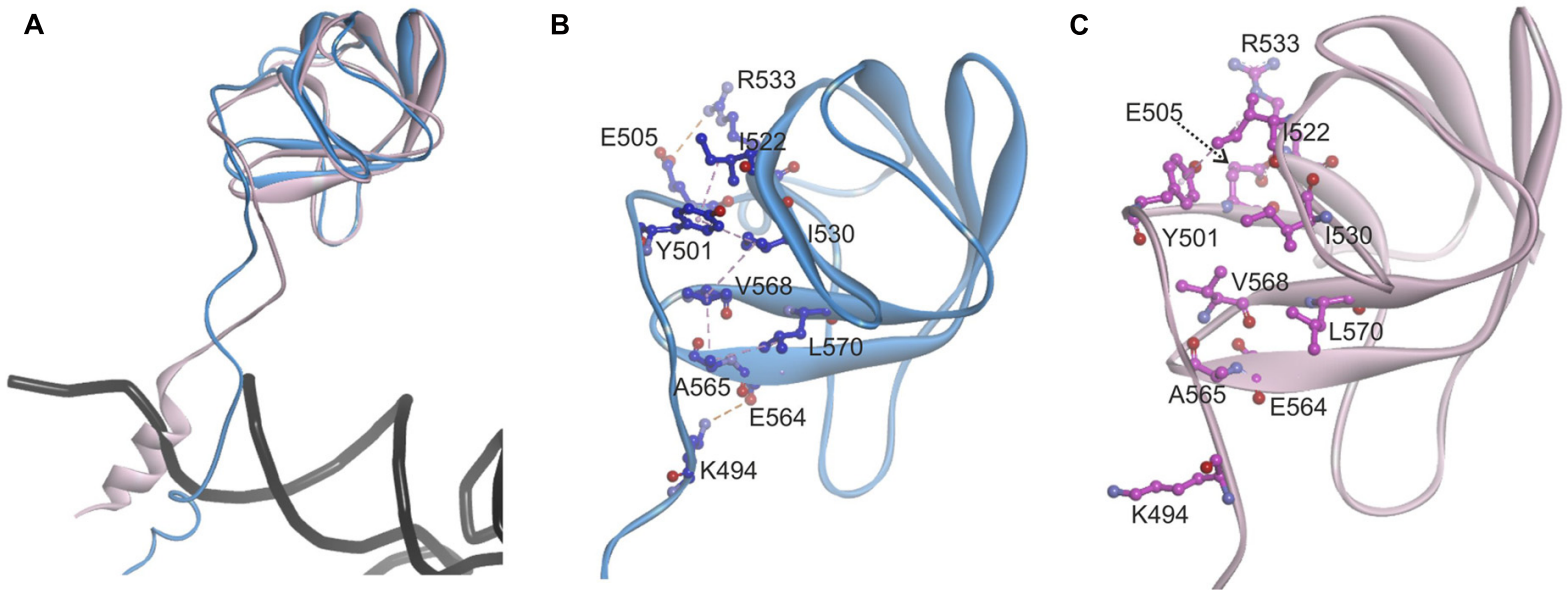
**Analysis of the contact surface between linker (“safety belt”) and β-barrel of the editing domain in ***A.**** fulgidus* AlaRS structure (3WQY), which was used as template for modeling. (A)** Superimposed chains A “closed” form (blue) and B “open” form (pink). Fragments encompassing residues K474–D589 are shown. For superimposition T582–V613 fragment was used as tether. **(B)** Residues from the linker (“safety belt”) interacting with the surface exposed residues on the β-barrel in “closed” conformation. Carbon atoms of interacting amino acid residues are in blue. **(C)** The position of the same residues in “open” conformation (carbon atoms in magenta). Hydrophobic interactions are marked with magenta dashed lines, electrostatic interactions with orange dashed lines.

**FIGURE 6:**
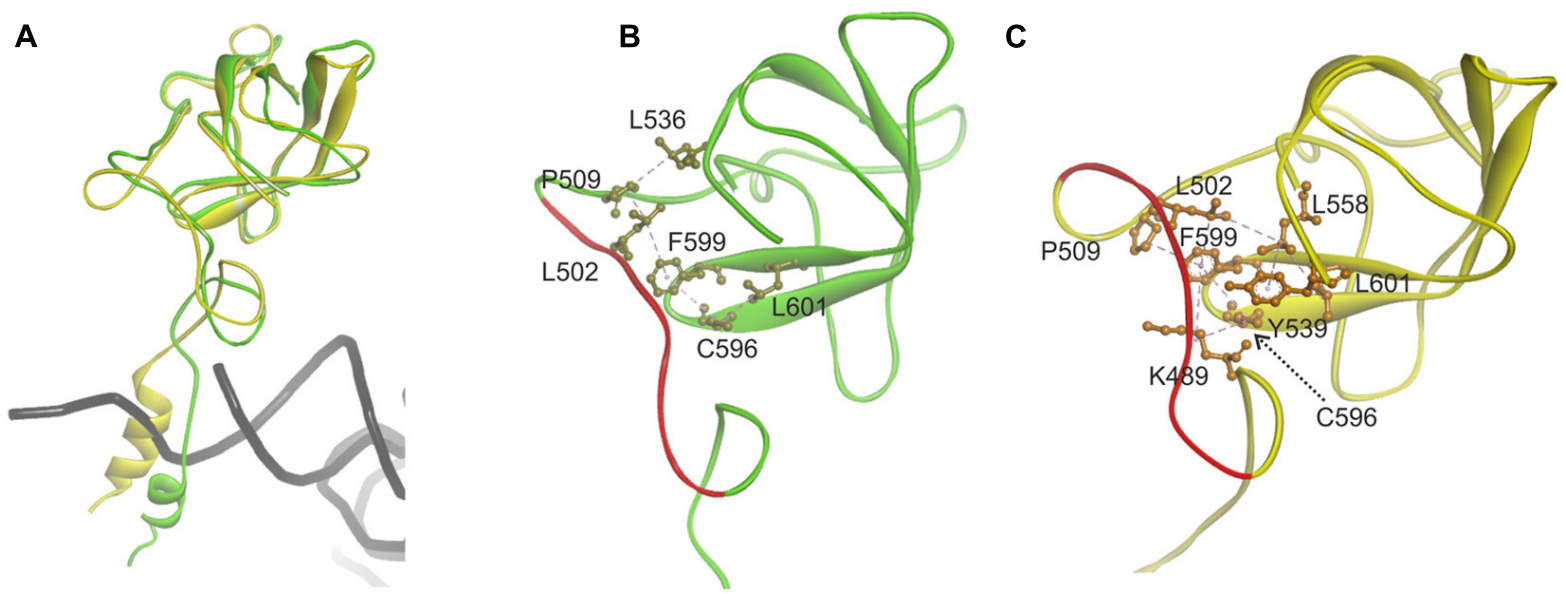
**Analysis of the contact surface between linker (“safety belt”) and β-barrel of the editing domain in modeled human mitochondrial AlaRS. (A)** Superimposed chains A “closed” form (green) and B “open” form (yellow). Fragments encompassing residues G468-D621 are shown. For superimposition Q615–L646 fragment was used as tether. **(B)** Interacting residues from the linker (”safety belt”) and surface exposed residues on the β-barrel in ”closed” conformation (shown in khaki). **(C)** Interacting residue in the “open” conformation (in orange). Hydrophobic interactions are marked with magenta dashed lines. Fragment L494–Q505 conserved between mitochondrial AlaRS homologs is marked with red.

Since the template structure of AlaRS of *A. fulgidus* was resolved as a dimer, with one subunit in a substrate bound “closed” state and the other in a substrate free “open” state (Naganuma et al., 2014), it was possible to use bacterial tRNA^Ala^ and alanyl-adenylate bound to the template structure (chain A in 3WQY) for docking into our model and explore intermolecular interactions within the tertiary complex (**Figure 4**). The homology modeling suggests that human mtAlaRS shares with *A. fulgidus* AlaRS the unique structural features inherent for all AlaRSs. Firstly, it has a large tRNA-binding cavity whereas other ARSs adopt cognate tRNAs on their surface. Secondly, all three domains are involved in the binding of tRNA undergoing aminoacylation. Previous studies have shown that tRNA binding and the rate of aminoacylation are enhanced by the C-terminal domain as it has the highest tRNA binding affinity (Guo et al., 2009a). The structure of *A. fulgidus* AlaRS revealed that the C-terminal globular subdomain holds the elbow of the L-shaped tRNA while the acceptor stem is fixed within the aminoacylation domain by concerted binding to the so-called “safety belt” (the linker between aminoacylation and editing domains), and to the tRNA recognition subdomain and the surface of editing core (Naganuma et al., 2014). We predict the same type of tRNA binding for aminoacylation also in the modeled human mtAlaRS (**Figure 4**). Close inspection of the *A. fulgidus* AlaRS structure as well as the modeled tertiary complex of mtAlaRS revealed that the “safety belt” folds along the surface of the β-barrel in the editing domain and establishes different interactions with its surface exposed residues in “open” and “closed” states (**Figure 5**). This suggests that the β-barrel may serve as a “buckle” for fastening the “safety belt” when the tRNA is bound in the aminoacylation site. In human mtAlaRS the interacting surfaces of the linker and β-barrel are comprised of different residues than in the template, but comparison of “open” and “closed” conformations shows rearrangements in their interactions upon tRNA binding (**Figure 6**). Interestingly, the linker has an amino acid sequence stretch, L494-Q505, which is highly conserved between mitochondrial homologs (**Figure 6**). Modeled structure shows that L502 from this stretch is involved in hydrophobic interactions predominantly with residues from β-hairpin (R592–A604) of the β-barrel. Since this motif is also very conserved between mitochondrial homologs and oriented toward the linker it is likely that these two fragments extend their interaction by establishing transient contacts upon sliding of the linker on the surface of the β-barrel during transition from “open” to “closed” conformations of the enzyme. Therefore, we propose that the editing domain of mtAlaRS, in addition to its proofreading activity of deacylating mischarged tRNAs, may also be involved in the aminoacylation activity of the enzyme by being involved in the binding of tRNA^Ala^ for aminoacylation and stabilizing position of its acceptor stem within the catalytic site.

### STRUCTURAL ANALYSIS OF *AARS2* MUTATIONS

**FIGURE 7:**
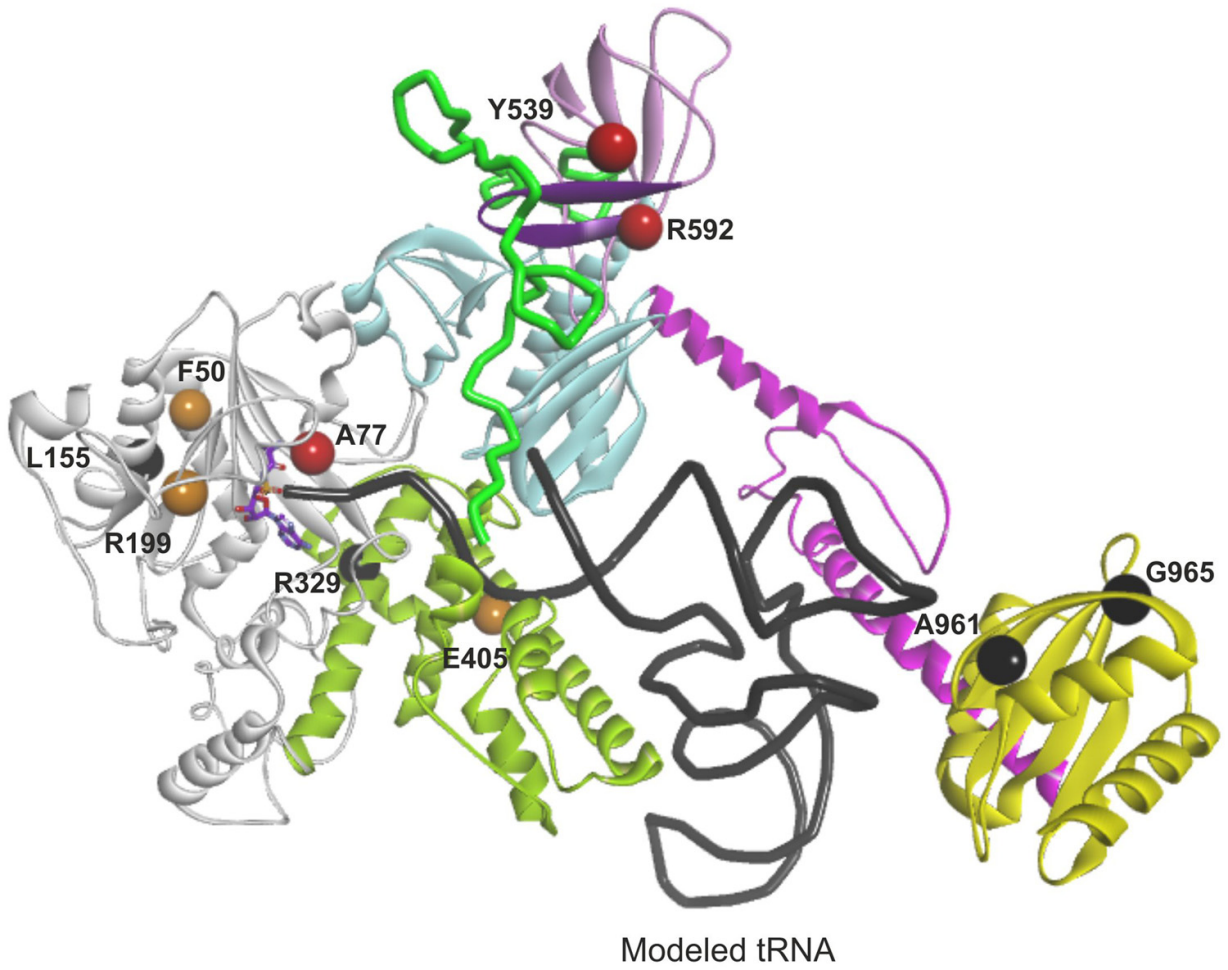
**Mapping and function predictions of *AARS2* mutations associated with cardiomyopathy and leukodystrophy.** Domains are color-coded as in **Figure 4**. Structural loss-of-function mutations are marked in black, mutations affecting substrate binding and resulting in severe reduction in aminoacylation activity are in red, and mutations resulting in moderate decrease of aminoacylation activity are in orange.

We utilized our full-length mtAlaRS homology model to localize all amino acid changes found in mtARS in patients with cardiomyopathy (L155R, R329H, Y539C, R592W, and A961V) and leukodystrophy (F50C, A77V, R199C, E405K, and G965R; **Figure 7**) to predict their effects on synthetase structure and function (**Table 3**). The missense mutations could be divided into three groups based on the effect on the aminoacylation activity: loss-of-function, severe, and moderate.

**Table 3:** Structural analysis of cardiomyopathy and leukodystrophy mutations in *AARS2* and assessment of their impact on the synthetase function based on modeled protein structure.

Amino acid change	Structural assessment	Functional impact on the enzyme	Phenotype C or L	In combination with
**Loss-of-function**	
L155R	Architectural residue maintaining structure of aminoacylation site; mutation impairs structural stability of the protein during folding	Loss-of-function mutation due to highly unstable protein	C	R592W
R329H	Structural residue involved in interaction and stabilization of two subdomains of the aminoacylation domain	Loss-of-function mutation due to highly unstable protein	C	R592W
A961V	Structural residue, involved in stabilization of globular subdomain of C-terminal domain, critical for architecture of tRNA binding surface	Loss-of-function mutation due to unstable protein and abolished tRNA binding	C	R592W
G965R	Structural residue, involved in stabilization of globular subdomain of C-terminal domain, critical for architecture of tRNA binding surface	Loss-of-function mutation due to unstable protein and abolished tRNA binding	L	E405K
**Severe**	
A77V	Catalytic residue; likely affects alanine binding	Results in either totally inactive enzyme or with little aminoacylation activity due to decreased affinity to alanine	L	R199C
Y539C	Surface exposed residue within the β-barrel subdomain of the editing domain, together with linker secures the position of tRNA within the aminoacylation site	Dramatic decrease of aminoacylation rate due to impaired tRNA binding and positioning of the 3′-end within the active site	C	R592W
R592W	Surface exposed residue within the β-barrel subdomain of the editing domain, together with linker secures the position of tRNA within the aminoacylation site	Dramatic decrease of aminoacylation rate due to impaired tRNA binding and positioning of the 3′-end within the active site	C	R592W, Y539C, R329H, L155R, A961V, truncating mutation
**Moderate**	
F50C	Architectural residue maintaining structure of aminoacylation site	Reduced rate of aminoacylation due to instability of alanine- and ATP-binding sites and impaired alanyl-adenylate formation	L	truncating mutation
R199C	Catalytic residue involved in ATP binding	Reduced rate of tRNA aminoacylation due to affected ATP-binding and impaired alanyl-adenylate formation	L	A77V, F131del, truncating mutations
E405K	Structural residue within the tRNA recognition subdomain of the aminoacylation domain	Partly reduced rate of tRNA aminoacylation due to structural instability in the tRNA recognition fold	L	G965R

*Almost all listed residues are invariant within AlaRSs, except R592. (C, cardiomyopathy; L, leukodystrophy)*.

#### Loss of function

The amino acid changes L155R, R329H, A961V, and G965R (residues L173, R345, A880, and V884 in *A. fulgidus*, respectively) are predicted to impair protein folding and stability resulting in loss of aminoacylation activity (black color-coded in **Figure 7**). Most of these residues are invariant between cytoplasmic, mitochondrial, and bacterial homologs of AlaRS and critical for maintaining the structure. Accordingly, the change L155R was recently modeled in yeast and had a deleterious effect on cell growth suggesting complete absence of aminoacylation activity (Dallabona et al., 2014). Residues A961 and G965 are important for the fold and stability of C-globular subdomain, and therefore for the architecture of the tRNA binding site. The C-terminal globular domain is separated from the main body of the enzyme, so its disruption may not result in overall misfolding. However, even if the aminoacylation and editing domains remain stable, disruption of the C-terminal domain’s crucial role in tRNA binding and orientation is likely to result in severely reduced rates of aminoacylation.

#### Severe

The A77V, Y539C, and R592W (residues A99, E525, and D561 in *A. fulgidus,* respectively) changes are not predicted to affect protein stability, as they are all surface exposed (red color-coded in **Figure 7**). However, each of these mutations is predicted to result in dramatic reduction of aminoacylation rate due to effects on substrate binding. In case of A77V (invariant residue in all AlaRSs) the binding and activation of alanine is likely to be affected. Y539C and R592W are predicted to impair tRNA binding and stabilization within the aminoacylation site by affecting interactions between the linker and β-barrel that we propose to be important for binding and securing acceptor stem of tRNA^Ala^ within the aminoacylation domain.

#### Moderate

The F50C, R199C, and E405K (residues F73, E202, and K416 in *A. fulgidus*, respectively) changes (orange color-coded in **Figure 7**) affect structural residues and are predicted to cause local structural disturbances within the aminoacylation domain leading to partial reduction of aminoacylation activity. The F50C mutant modeled in yeast showed partial reduction of tRNA^Ala^ charging and protein instability, which became noticeable only at a higher temperature (Dallabona et al., 2014). These results are in line with our structural predictions.

**FIGURE 8:**
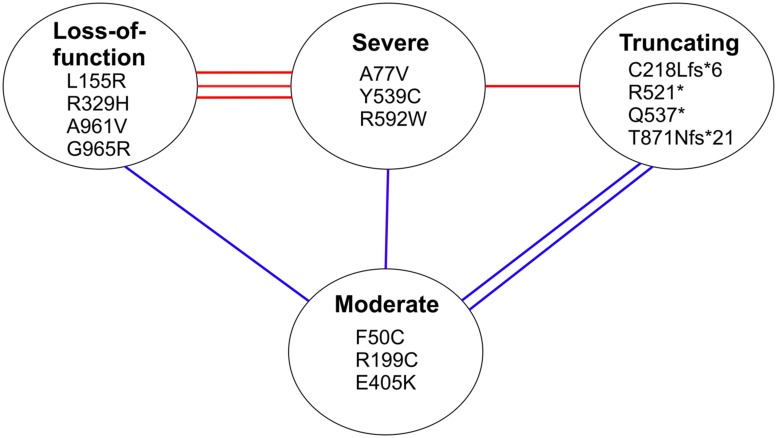
**Classification of mutations based on structural predictions.** The lines between mutation categories connect the compound heterozygous mutations identified in individual cardiomyopathy patients (with red lines), and leukodystrophy patients (with blue lines). The three categories at the top are predicted to have a severe effect on the aminoacylation function of the synthetase, whereas the mutations in the lower category are predicted to retain partial catalytic activity.

In conclusion, our analysis of the homology model predicts that the missense mutations in the first two categories, affecting overall structure and substrate binding, severely compromise the aminoacylation by the synthetase, which is also true for the truncating mutations, whereas the missense mutations in the third category result in reduced function but with some retained aminoacylation activity. Combining the data of the compound heterozygous mutations in each patient clearly shows that all patients with leukodystrophy have combinations where one allele is in one of the two severe categories or is a truncating mutation and the other allele is in the third, moderate category, whereas all mutations of the cardiomyopathy patients are severe (**Figure 8**).

## DISCUSSION

The tissue-specificity of mitochondrial diseases is puzzling. The pathogenic mechanisms behind the tissue-specific manifestations are likely to involve cell type-specific mitochondrial functions and metabolite requirements, in addition to defects of ATP production by oxidative phosphorylation (Nunnari and Suomalainen, 2012). Differential basal mRNA expression levels of mtARSs have been suggested to contribute to the tissue-specificity, as brain, muscle, and heart were found to have low levels of mtARS mRNAs compared to other tissues (Florentz et al., 2013). The example of *AARS2*, however, shows that mutations in the same mtARS can also give rise to highly different tissue-specific diseases.

Our previous hypothesis for the cardiomyopathy-related R592W was that it affected tRNA binding in the editing domain and thus interfered with the proofreading of mischarged tRNAs. The homology modeling of human mtAlaRS, based on the new resolved structure of AlaRS of *A. fulgidus*, suggested instead that all domains of the synthetase, including the editing domain, have an important role in tRNA binding for aminoacylation. We speculate whether this unique way of cooperative tRNA binding for aminoacylation in AlaRSs might explain why the mtAlaRS has a preserved editing domain whereas most other mitochondrial ARSs do not. For example, editing activity was lost during the evolution of mtPheRS (Roy et al., 2005; Elo et al., 2012), and the editing active site of mtLeuRS is not operational (Lue and Kelley, 2005). Although the change at residue R592 does not appear to affect the editing active site, we cannot rule out an effect on editing activity, but we propose that the crucial consequence is on tRNA binding for aminoacylation.

We suggest that the two distinct phenotypes caused by *AARS2* mutations are simply caused by differential effects on aminoacylation, with cardiomyopathy resulting from highly severe reduction in aminoacylation activity, and leukodystrophy resulting from only partial reduction in activity. This conclusion could be reached only by careful analysis of the combined mutations found in the patients. The clinical feature of the cardiomyopathy patients was the heart defect, although OXPHOS defects could be seen also in muscle and brain. This suggests that the patients had a combined tissue-involvement that can be expected when mitochondrial protein synthesis is significantly compromised, and that other tissues would have also suffered, had the patients lived longer.

We have previously proposed that severe defects of mitochondrial translation manifest in the heart immediately or soon after birth (Ahola et al., 2014), most often leading to early death. However, those few patients who survived the early severe cardiac disease (Carroll et al., 2013; Haack et al., 2013; Ahola et al., 2014) stabilized for their heart function after a few years of age, but later tended to develop a brain disease. These findings suggest a special requirement for mitochondrial protein synthesis in the heart in the early life, which can be partially compensated for after a critical window of sensitivity, only to be followed by manifestations in the sensitive organ of later childhood-adulthood years, the brain. This hypothesis is also supported by our current findings: the severe *AARS2* mutations manifested in the heart soon after birth – or even in late pregnancy – whereas the partial reduction of the synthetase function manifested from 2 to 40 years of age in the brain (Dallabona et al., 2014).

In the future, *in vivo* studies on the partial retained activity of the leukodystrophy mutations – sufficient for heart function but limiting for brain function during later years – are warranted, to get further insight into the particular events taking place in the heart and in the brain, constituting ‘the narrow permissive window for disease manifestation’ (Steenweg et al., 2012; Ahola et al., 2014). The roles of amino acid availability and varying mtARS expression levels in different developmental stages of tissues, as well as the indispensable level of mitochondrial protein synthesis in each stage and cell type, are of interest.

## Conflict of Interest Statement

The authors declare that the research was conducted in the absence of any commercial or financial relationships that could be construed as a potential conflict of interest.
